# Absence of modulatory effects of 6Hz cerebellar transcranial alternating current stimulation on fear learning in men

**DOI:** 10.3389/fnhum.2023.1328283

**Published:** 2024-01-09

**Authors:** Sarah Johanna Schellen, Philip Zeidan, Thomas M. Ernst, Andreas Thieme, Seyed Ali Nicksirat, Christian J. Merz, Michael A. Nitsche, Fatemeh Yavari, Dagmar Timmann, Giorgi Batsikadze

**Affiliations:** ^1^Department of Neurology and Center for Translational Neuro- and Behavioral Sciences (C-TNBS), University Hospital Essen, Essen, Germany; ^2^Department of Cognitive Psychology, Faculty of Psychology, Institute of Cognitive Neuroscience, Ruhr University Bochum, Bochum, Germany; ^3^Department of Psychology and Neurosciences, Leibniz Research Center for Working Environment and Human Factors, Dortmund, Germany; ^4^German Center for Mental Health (DZPG), Bochum, Germany

**Keywords:** fear conditioning, transcranial alternating current stimulation, aversive conditioning, associative learning, pavlovian conditioning, non-invasive brain stimulation

## Abstract

Fear is a vital defense mechanism to potential threats, which is influenced by the cerebellum. While the cerebellum’s role in acquiring fear responses is well understood, limited knowledge exists about its involvement in fear extinction. In this study, we investigated the effects of cerebellar theta band transcranial alternating current stimulation (ctACS) administered during fear extinction training, based on previous evidence from animal studies suggesting a role of cerebellar theta oscillations in associative memory formation. To this end, thirty-seven healthy right-handed male participants were recruited for a two-day differential fear renewal paradigm. On day 1, they underwent acquisition training in context A followed by extinction training in context B. On day 2, recall was tested in contexts A and B. One group of participants received ctACS in the theta band (6 Hz) during extinction training. The other group received sham ctACS. Although both groups demonstrated the ability to recall previously learned fear and distinguish between low and high threat stimuli, no significant differences were observed between the ctACS and sham groups, indicating that ctACS at this theta frequency range did not impact extinction and recall of previously acquired fear in this study. Nevertheless, using ctACS could still be useful in future research, including brain imaging studies, to better understand how the cerebellum is involved in fear and extinction processes.

## Introduction

Fear, a vital defense mechanism, safeguards both animals and humans from danger. Fear modulation, with its complex processes, such as fear acquisition, extinction and recall, plays a fundamental role in shaping emotional responses to potential threats. Yet, maladaptive fear responses can lead to anxiety and stress-related disorders. These conditions are believed to be caused by abnormal fear conditioning and difficulties in recalling extinction (Pitman, 1988; VanElzakker et al., 2014). Consequently, investigating strategies to modulate fear is a critical area of research in neuroscience, with the aim of developing effective interventions for fear-related conditions.

Advancements in non-invasive brain stimulation (NIBS) techniques offer opportunities to explore fear regulation and potential therapies. Studies on post-traumatic stress disorder (PTSD) patients revealed deficits in extinction recall, linked to reduced ventromedial prefrontal cortex (vmPFC) volume and activity during fear extinction (Giustino and Maren, 2015; Gonzalez and Fanselow, 2020, for review). To enhance extinction learning, researchers attempted vmPFC stimulation, but the results have been inconclusive. Some studies reported improvement in extinction learning and safety learning, while others showed no effect or even adverse outcomes (Adams et al., 2020; Marković et al., 2021, for review). One possible reason for the inconsistency might be the limited ability to directly stimulate the vmPFC, leading some of these studies to target alternative brain areas, such as the prefrontal or supraorbital cortices in order to indirectly reach the vmPFC, which may produce varied effects on fear learning. As an alternative, targeting the cerebellum with NIBS could be a promising approach. While traditionally associated with motor coordination, the cerebellum is also involved in cognitive and emotional processes, including the acquisition and retention of conditioned fear responses (Hwang et al., 2022; Doubliez et al., 2023, for review). Recent studies indicate its engagement in fear learning, possibly through predictions and prediction errors (Ernst et al., 2019; Batsikadze et al., 2022).

The research involving fear conditioning and NIBS mainly focuses on transcranial direct current stimulation (tDCS). However, transcranial alternating current stimulation (tACS) has also been widely explored as a promising alternative in the motor and cognitive domains (Wessel et al., 2023, for review). tACS is suggested to operate based on the entrainment theory, i.e., it can synchronize and amplify intrinsic neuronal oscillations (Tavakoli and Yun, 2017, for review). The choice of the theta frequency band (4–7 Hz) for our study is grounded in its significance for creating temporal associations among sensory stimuli and its fundamental role in processes like associative learning and motor adaptation (Chen et al., 2016, for review; Herweg et al., 2020; Tzvi et al., 2022). Cerebellar theta oscillations may synchronize with hippocampal theta during trace conditioning, suggesting a role in associative memory formation (Hoffmann and Berry, 2009). Spontaneous cerebellar theta activity is linked to successful extinction of conditioned eyeblink responses in guinea pigs (Wang et al., 2014), while reduced cerebellar theta activity following the conditioned stimulus (CS) correlates with the subsequent spontaneous recovery of previously extinguished responses (Wang et al., 2019). Our study aimed to investigate how cerebellar tACS (ctACS) in the theta frequency range (specifically, 6 Hz) affects fear modulation. We used a two-day fear learning paradigm based on the one published by Batsikadze et al. (2022), originally introduced by Milad et al. (2007). We administered either 6 Hz or sham ctACS during extinction training on day 1, examining its impact on recall of learned fear on day 2.

## Methods

### Subjects

This study included only men, as menstrual cycle and oral contraceptives can affect fear learning differently (Merz et al., 2018). For this current study, we determined the necessary sample size using G*Power software (Faul et al., 2009). To achieve a medium effect size [*f* = 0.25, (Cohen, 1988)], 40 participants were divided into two groups. This calculation was based on a significance level α = 0.05, an assumed correlation *r* = 0.35 among repeated measurements, and a desired statistical power (1 – β) of 0.9. We recruited a total of 45 young and healthy right-handed men, aged 24.38 ± 4.06 years. None of the participants had neurological or neuropsychiatric disorders or took centrally acting medication. They were naïve to both brain stimulation and fear learning procedures. Prior to the experiment, participants were examined by experienced neurologists (AT, SAN) and their depression, anxiety, and stress levels were assessed using the DASS-21 questionnaire (Henry and Crawford, 2005; Norton, 2007). We excluded three participants with moderate or higher depression, anxiety, or stress scores on the DASS-21 questionnaire (Lovibond and Lovibond, 1995) and later five more due to technical issues during data acquisition. As a result, 37 participants (aged 24.05 ± 3.72 years) were included in the final data analysis. Their DASS-21 scores fell within the normal-to-mild range: median depression score of 2 (interquartile range - IQR 0 - 6, range 0 - 12), median anxiety score of 2 (IQR 0 - 4.5, range 0 - 8), and median stress score of 5 (IQR 2 - 12, range 0 - 16). Additionally, participants were asked to avoid alcohol consumption for at least 24 h before the experiment. The study was approved by the Ethics Committee of the University Hospital Essen and conforms to the principles laid down in the Declaration of Helsinki. Informed consent was obtained from all participants, and they were compensated with 70 Euros for their participation.

Participants were split into two stimulation groups: verum (18 participants) and sham (19 participants). The verum group received 2 mA (peak-to-peak) 6 Hz ctACS for 15 min, while the sham group received sham ctACS for 30 s. ctACS was administered using a battery-driven constant current stimulator (DC-Stimulator Plus, neuroConn GmbH, Ilmenau, Germany) and a pair of rubber electrodes (5 × 7 cm^2^) with conductive paste (Ten20, Weaver) applied, started two minutes before the beginning of the extinction training and continued until its completion. The target electrode was placed vertically over the right cerebellar cortex (centered 2 cm below and 3 cm lateral to the inion). This selection was based on a previously demonstrated modulatory effect in an eyeblink conditioning paradigm (Zuchowski et al., 2014). The non-target electrode was positioned horizontally over the right deltoid muscle (Batsikadze et al., 2019). The current was ramped up and down for 20 s at the start and the end of stimulation. The *fade-in−short stimulation−fade-out* approach for sham tDCS was employed in our study. This technique involves gradually increasing the intensity of the stimulation (*fade-in*, 20 s), delivering a brief period of stimulation with target intensity (*short stimulation*, 30 s), and then gradually reducing the intensity back to zero (*fade-out*, 20 s). This procedure is conventional and has been shown to be indistinguishable from real stimulation in terms of the persistence of sensations on the skin associated with actual stimulation (Ambrus et al., 2012). However, it is also intentionally brief to prevent unwanted after-effects (Dissanayaka et al., 2018). Both the experimenter and the participants were unaware of the type of stimulation being administered. Double blinding was accomplished using the study mode of the stimulator. Pre-assigned five-digit codes were entered into the device, initiating either the active or sham protocol.

### Experimental procedures

**FIGURE 1:**
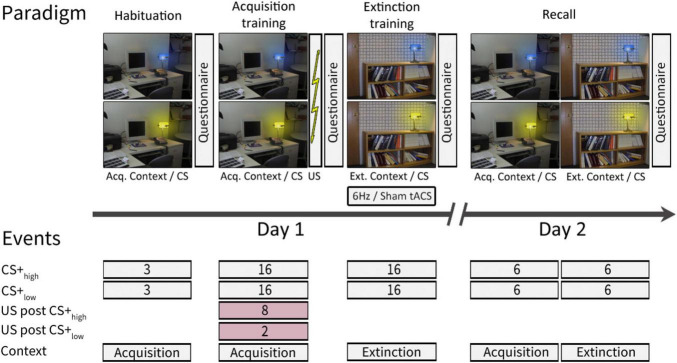
Experimental paradigm [adapted from Batsikadze et al. (2022)]. On day 1, habituation and acquisition training were performed in the acquisition context, while extinction training took place in the extinction context. On day 2, recall trials were presented in both the acquisition and extinction contexts. Contexts were represented by a photography of either a desk or a bookshelf. The CSs were represented by the same desk lamp shining either in blue or yellow color. For further details see text. This study follows a modified version of the experimental paradigm initially introduced by Milad et al. (2007) and described in Batsikadze et al. (2022). ctACS, cerebellar transcranial alternating current stimulation; CS, conditioning stimulus; US, unconditioned stimulus; Acq. Context, context presented during acquisition training, Ext. Context, context presented during extinction training.

The experiment was conducted over two consecutive days following a paradigm initially introduced by Milad et al. (2007) and described in Batsikadze et al. (2022), involving one CS+ and one CS−. The original plan thus aimed for a 62.5% reinforcement rate for the CS+ and no reinforcement for CS−, meaning that 10 out of 16 acquisition training CS+ trials should have been paired with a US. However, due to a coding error that was discovered after completing the data collection, two CS− trials in the late acquisition training phase were mistakenly reinforced. This unintentionally transformed the neutral stimulus into a negative one with a low reinforcement rate. Consequently, differential fear conditioning was conducted with two conditioned stimuli (CS+) with different reinforcement rates (CS+_*high*_ and CS+_*low*_, respectively). In this altered scenario, instead of the initially intended 10, 8 out of 16 CS+ trials were reinforced, resulting in a 50% reinforcement rate for CS+_*high*_ and rather than none, 2 out of 16 CS− trials were paired with a US, resulting in a 12.5% reinforcement rate for CS+_*low*_. In unreinforced trials (CS+ only), the CS+ was not followed by the US (Figure 1).

Day 1 comprised three phases: “habituation” (consisting of 3 CS+_*high*_ only and 3 CS+_*low*_ only trials, presented in the acquisition context), “fear acquisition training” (including 8 paired CS+_*high*_/US, 2 paired CS+_*low*_/US, 8 CS+_*high*_ only, and 14 CS+_*low*_ only trials, presented in the acquisition context), and “extinction training” (comprising 16 CS+_*high*_ only and 16 CS+_*low*_ only trials, presented in the extinction context). On Day 2, there was a recall phase (including 12 CS+_*high*_ only and 12 CS+_*low*_ only trials, evenly distributed between the acquisition and extinction contexts). The order of trial types in each phase was pseudo-randomized. A neutral gray background with a black cross image was shown before the first context picture onset and during the initial two minutes of ctACS prior to extinction training.

During the experiment, acquisition and extinction contexts were represented by two office space photographs: one featuring a desk and the other a bookshelf, both including an identical desk lamp. The lamp emitted blue or yellow light, serving as the CS. Each CS presentation lasted 8 s, followed by an intertrial interval of 18.52 ± 1.95 s. In reinforced trials, a 100 ms aversive US was presented after 7.9 s, synchronized with the termination of the CS. The context image was continuously displayed, including between CS presentations. The allocation of contexts and CS colors was pseudo-randomly balanced among participants. During the recall phase, if two consecutive events were presented in different contexts, the context picture would change 2 ± 0.6 s before the onset of the second CS.

The trial types in each phase were pseudorandomized. For each phase, we created a sequence of events while following two specific rules: we ensured that no more than two consecutive trial events of the same type were presented, and we maintained an equal number of CS+_*high*_ and CS+_*low*_ events, as well as events presented in the acquisition and extinction contexts (where applicable, e.g., recall) in both the first and second halves of the phase. The same sequence was used for each participant for habituation, acquisition training, and extinction training phases. In the recall phase, two nearly identical sequences were used, counterbalanced among the subjects. These sequences differed in the order of the CS+ stimuli for the first and third trials, which were presented in the acquisition context: either 1*^st^* CS+_*high*_ and 3*^rd^* CS+_*low*_ or 1*^st^* CS+_*low*_ and 3*^rd^* CS+_*high*_.

A brief electrical stimulation consisting of a train of four consecutive 500 μs current pulses was applied to the left shin as the aversive US. The stimulation intensity was individually adjusted by participants on a nine-point Likert-scale of 1 to 9, ranging from “*not unpleasant”* to “*very unpleasant*,” until it reached a level rated as 8. This adjustment was made at the beginning of the experiment on day 1. The intensity of the aversive US was 2.16 ± 1.8 mA, ranging from 0.5 mA to 7.5 mA. To counteract habituation and prevent weakening of the conditioned responses (Inoue et al., 2020), 20% was added to each participant’s individual thresholds. The mean added current was 0.43 mA ± 0.36 mA, and this adjusted intensity 2.6 ± 2.17 mA was kept constant throughout the experiment. To ensure precise placement on day 2, the electrode position on the skin was marked using a permanent marker on day 1.

Skin conductance responses (SCRs) were recorded throughout the experiment using two SCR electrodes placed on the hypothenar of the left hand. After each phase of the experiment, participants completed questionnaires to assess their subjective experience. They rated the valence, arousal, fear, and expectancy of an US associated with viewing images of the CS+_*high*_ and CS+_*low*_ on a nine-point Likert scale. The scale ranged from “*very pleasant”* to “*very unpleasant”* for valence, “*very calm”* to “*very nervous”* for arousal, “*not afraid”* to “*very afraid”* for fear, and “*US not expected*” to “*US expected*” for US expectancy.

After fear acquisition training participants were asked to provide feedback on their perception of the aversive unconditioned stimulus (US). They rated the intensity of the last US on a nine-point Likert scale, ranging from “*not unpleasant*” to “*very unpleasant*”. In addition, participants were requested to assess the probability of the US occurring after each conditioned stimulus (CS) presentation using a 0–100% scale with 10% intervals.

Participants were also asked to complete a questionnaire before and after the stimulation to assess potential side effects. The questionnaire [adapted from Brunoni et al. (2011)], included items related to headache, neck pain, back pain, blurred vision, scalp irritation, scalp tingling, scalp itching, increased heartbeat, burning sensation, hot flashes, vertigo, sudden mood change, fatigue, and phosphenes. Participants rated the intensity of these side effects on a Likert scale ranging from 1 (“*absent*“) to 9 (“*strong*”).

The questions and rating scales were presented on a computer screen, and participants used a button box with their right hand to provide their responses.

### Analysis and statistics

SCRs were recorded using a data acquisition station (MP160, BIOPAC Systems Inc., Goleta, CA) with a gain of 10 μS/V and a sampling rate of 1 kHz. To reduce noise, the SCRs were low-pass filtered at 10 Hz using a hardware filter (EDA100C-MRI module, BIOPAC Systems Inc., Goleta, CA). MATLAB software (Release 2019a, RRID:SCR_001622, The MathWorks Inc., Natick, MA) was used for semi-automated peak detection. SCRs were defined as the maximum trough-to-peak amplitude of any peak meeting specific criteria, including a minimum amplitude of 0.01 μS and a minimum rise time of 500 ms (Boucsein et al., 2012), starting within a time interval from 1 to 8 s after CS onset. Trials not meeting the criteria were scored as zero and included in subsequent analysis (Pineles et al., 2009).

The raw SCR amplitudes were normalized using a logarithmic transformation [ln(1 + SCR)] (Boucsein et al., 2012; Braithwaite et al., 2013). Non-parametric statistical analysis was conducted due to the non-normal distribution of the data and residuals (Shapiro-Wilk test, *p* < 0.05), using the ANOVAF option in PROC Mixed procedure in SAS (SAS Studio 3.8, SAS Institute Inc., Cary, NC, USA) and the *nparLD* R package.^1^ These methods are recommended for handling skewed distributions, outliers, or small sample sizes. To enhance the reliability of the analysis, an ANOVA-type statistic (ATS) was used with the denominator degrees of freedom set to infinity (Brunner et al., 2002; Shah and Madden, 2004; Noguchi et al., 2012) as the use of finite denominator degrees of freedom can result in increased type I errors (Bathke et al., 2009). The ATS were applied to each phase, using SCR as dependent variable, stimulus (CS+_*high*_, CS+_*low*_), context (only in recall: acq. context, ext. context) and trial as within-subject factors, and group (verum, sham) as a between-subject factor, along with their interactions. *Post hoc* comparisons were performed using least square means tests.

To account for the observed group differences in habituation rates during the acquisition training phase, we calculated the differential SCR (SCR_*diff*_) by subtracting the SCR to CS+_*low*_ from the SCR to CS+_*high*_ for each respective trial presented in the same context (Ganella et al., 2017; Albayrak et al., 2023). This allowed us to quantify the differential response to the conditioned stimuli. The ATS for repeated measures were applied to each phase, using SCR_*diff*_ as dependent variable, trial and context (only in recall) as within-subject factors, and group (verum, sham) as a between-subject factor, along with their interactions.

The questionnaires were analyzed using the ATS for repeated measures. The respective rating was used as the dependent variable, stimulus (CS+_*high*_, CS+_*low*_) and time (prior to and post-fear acquisition training, post-extinction training, post-recall) as within-subject factors, and group (verum, sham) as a between-subject factor, along with their interactions.

Similarly, the ratings for possible side effects were analyzed using the ATS for repeated measures, using the respective rating as the dependent variable, time (prior to and post ctACS) as a within-subject factor, and group (verum, sham) as a between-subject factor, along with their interactions.

## Results

### Skin conductance responses (SCRs)

*Habituation phase (day 1)*: participants showed higher mean SCR amplitudes for CS+_*high*_ compared to CS+_*low*_, likely due to CS+_*high*_ being presented first. The ATS revealed significant main effects of Stimulus (CS+_*high*_ vs. CS+_*low*_; *F*_1_ = 21.82, *p* ≤ 0.001) and Trial (Trials 1–3; *F*_2_ = 10.00, *p* ≤ 0.001), indicating differences in SCR amplitudes between stimuli and across trials, respectively. Initial trials exhibited significantly higher SCR amplitudes compared to subsequent habituation trials (both *p* values ≤ 0.001, least squares means test), with no significant differences between the second and third trials (*p* = 0.586, least squares means test).

**FIGURE 2:**
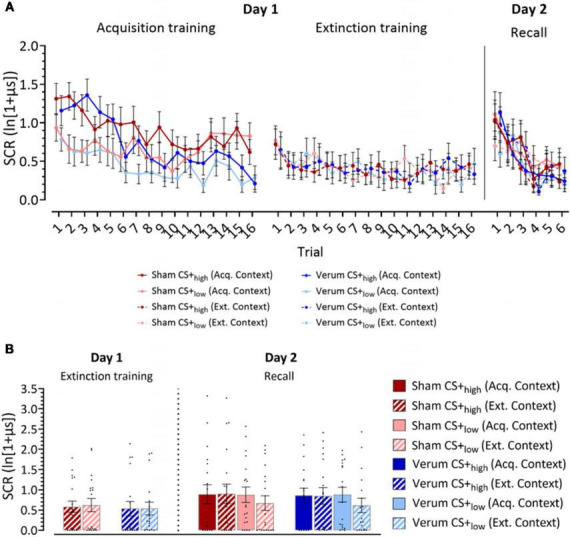
Log-transformed skin conductance response (SCR) amplitudes. **(A)** Mean SCR values for individual trials for acquisition training, extinction training and recall phases. **(B)** Recall of learned fear responses at the beginning of extinction training and recall. Panel **(A)** filled dots represent the mean values for individual trials for acquisition training, extinction training and recall phases. Solid lines connect mean values of trials presented in the acquisition context, while dotted lines connect mean values of trials presented in the extinction context. Dark colors represent CS+_*high*_, light colors represent CS+_*low*_. Panel **(B)** the figure shows mean SCRs averaged from the initial two trials of each phase presented in the same context. Individual responses are indicated by dots. Full bars–trials shown in the acquisition context, striped bars–trials shown in the extinction context. Error bars indicate S.E.M. Blue colors, verum, red colors, sham; CS, conditioning stimulus; Acq. Context, context presented during acquisition training; Ext. Context, context presented during extinction training.

*Acquisition phase (day 1)*: Both groups learned to differentiate the CS+_*high*_ from the CS+_*low*_ in the acquisition phase with significantly higher SCR amplitudes toward the CS+_*high*_ compared to the CS+_*low*_ (Figure 2A; Supplementary Table 1). SCR amplitudes were expected to decline in late compared to early acquisition, with SCR habituation being a common finding in fear conditioning studies (Merz et al., 2016; Inoue et al., 2020; Batsikadze et al., 2022). This was predominantly observed in the verum group. The ATS revealed significant main effects of Stimulus (CS+_*high*_ vs CS+_*low*_; *F*_1_ = 35.94, *p* ≤ 0.001), Trial (Trials 1–16; *F*_8_._62_ = 7.25, *p* ≤ 0.001), Stimulus × Trial (*F*_1_ = 2.74, *p* = 0.005) and the Group × Trial (*F*_1_ = 2.46, *p* = 0.015). Other main effects and interactions were not significant (all *p* values ≥ 0.139). *Post hoc* analysis of Group × Trial interaction revealed significantly higher SCRs toward both CSs in the sham group in trials 12, 15 and 16. *Post hoc* analysis of the Trial × Stimulus interaction revealed significantly higher SCRs toward CS+_*high*_ in trials 1, 2, 3, 4, 5, 6, 7, 10 and 12 compared to CS+_*low*_ (all *p* values ≤ 0.017).

*Extinction phase (day 1):* The ATS revealed a close-to-significant main effect of Trial (*p* = 0.056), which can be attributed to the initial fear extinction training trials eliciting a higher response. However, there were no other significant main effects or interactions (all *p* values ≥ 0.445, Figure 2A; Supplementary Table 1).

*Recall phase (day 2):* During early recall trials, higher SCRs were observed in both CS+_*high*_ and CS+_*low*_ stimuli compared to the late trials in both groups. The ATS revealed a significant main effect of Trial (Trials 1–6: *F*_2_._9_ = 13.250, *p* ≤ 0.001), but no other significant main effects or interactions (all *p* values ≥ 0.160). Pairwise comparisons showed that SCRs in trial 1 were significantly higher than in trials 2–6 (all *p* values ≤ 0.036, least squares means test), and SCRs in trial 2 were significantly higher than in trials 3–6 (all *p* values ≤ 0.003, least squares means test; Figure 2A; Supplementary Table 1).

### Initial trial analysis

Separate analyses were conducted on the first two extinction and recall trials to assess retrievals of learned fear (initial extinction trials) and learned extinction/spontaneous recovery (initial recall trials) (Kalisch et al., 2006; Inoue et al., 2020). To that end, SCRs from the first two trials of the extinction training with the same CS were averaged within their respective blocks. In recall, SCRs from the first two trials with the same CS presented in the same context were averaged into blocks.

*Extinction training (day 1).* The ATS revealed no significant main effects or interactions in the first extinction block (all *p* values ≥ 0.288; Figure 2B; Supplementary Table 1).

*Recall (day 2).* In the analysis of the SCRs in the first recall block, a close-to-significant main effect of Stimulus (*F*_1_ = 3.48, *p* = 0.062) and a significant Stimulus x Context interaction (*F*_1_ = 6.05, *p* = 0.014) were observed. *Post hoc* analysis using least squares means test revealed significant differences between CS+_*low*_ (acq. context) and CS+_*low*_ (ext. context) (*p* = 0.028), as well as between CS+_*high*_ (ext. context) and CS+_*low*_ (ext. context) (*p* = 0.007). Close-to-significant differences were also revealed between CS+_*high*_ (acq. context) and CS+_*low*_ (ext. context) (*p* = 0.064). CS+_*low*_ (ext. context) elicited the lowest SCR responses compared to other stimulus-context combinations (Figure 2B; Supplementary Table 1).

The results of the differential skin conductance responses (SCR_*diff*_) and questionnaire analysis are presented in the Supplementary material.

## Discussion

Our results indicate that participants successfully formed and recalled fear memories, while also differentiating previously learned low and high threat stimuli. However, the application of 6 Hz theta ctACS during extinction training did not modulate the process of extinction learning and recall.

The findings in our study are in line with other studies using non-invasive cerebellar NIBS techniques, which also reported no significant effects on various learning paradigms (Jalali et al., 2017; Liew et al., 2018; Mamlins et al., 2019; Rauscher et al., 2020; Nguemeni et al., 2021). The complexity of the cerebellar cortex’s gyral folding can lead to diverse polarization profiles at different sites during stimulation, resulting in varied global effects of cerebellar NIBS. This variability could potentially be limiting in achieving robust effects (van Dun et al., 2016; Benussi et al., 2023, for review).

However, other reasons may also contribute. Firstly, in our current study, the lack of detectable modulatory effects of ctACS on fear learning does not necessarily rule out cerebellar activity modulation. The complexity of our experimental paradigm could be a factor. Recent fMRI study has provided evidence of the cerebellum’s engagement in fear extinction training and processing fear- and safety-related information (Batsikadze et al., 2022). It is possible that the partial reinforcement of both CSs with an US led to very similar cerebellar neural activity patterns for both CS+s, making their modulation by 6Hz ctACS hard to detect, especially without a neutral (i.e., safe) stimulus for comparison. The lack of a neutral stimulus may have prevented the observation of more subtle stimulation effects. Moreover, the complexity of our paradigm involving two distinct images of office spaces as contexts, varied-colored lights as CSs could have affected the strength of learned associations. Similar absence of group differences has also been observed in a recent behavioral study of our group with a comparable paradigm (Albayrak et al., 2023). For a clearer understanding of ctACS effects on fear learning processes, future studies should consider simpler fear conditioning paradigms. These could involve CSs with consistent reinforcement rates, a neutral stimulus and a stable neutral context throughout the experiment. Furthermore, extinction training has been performed directly following acquisition training (i.e., immediate extinction) and consolidation of learned fear was likely not completed. Immediate extinction tends to result in a stronger return of fear (fear renewal) (Merz et al., 2016), and this phenomenon might have obscured the effects of ctACS on extinction training. Therefore, using an experimental paradigm with delayed extinction, where acquisition training, extinction training, and recall occur on different days of the experiment, might also be beneficial in future studies.

Secondly, the absence of ctACS after-effects in our study could also be attributed to the use of 2 mA peak-to-peak intensity, which effectively applies 1 mA zero-to-peak current intensity to the cerebellum. In a modeling study by Rampersad et al. (2014), it was reported that cerebellar tDCS resulted in a significant amount of shunting. As a result, the authors suggested that a larger input current of 2 mA, rather than 1 mA, should be utilized to achieve effective electric fields in the cerebellum. Additionally, when using extracephalic reference electrodes for NIBS, some authors have recommended to adjust intensity based on inter-electrode distance, considering current flow complexities to achieve comparable after-effects (Moliadze et al., 2010). Taken together, the current intensity applied in our study might not have been sufficient to effectively entrain cerebellar oscillations and modulate fear learning. Using stronger intensities (e.g., 1.5 or 2 mA zero-to-peak) might be essential to explore the potential of ctACS in future research.

Finally, while prior studies provide some support for the involvement of theta frequencies in eyeblink conditioning (Wang et al., 2014, 2019), our choice of a 6 Hz stimulation frequency might not have been the best fit for the complex processes of fear conditioning and extinction learning. It is possible that these processes involve neural mechanisms better aligned with frequencies outside of 6 Hz. For instance, as suggested by Urrutia Desmaison et al. (2023), the cerebellum appears to regulate 4 Hz oscillations between the cortex and thalamus during the retrieval of fear memories, potentially affecting the fear extinction process. Additionally, in the context of cerebello-hippocampal interaction the synchronization of Purkinje cell activity in both Crus I and Lobulus Simplex with the medial prefrontal cortex and hippocampal CA1 occurs in the delta (0.5–4 Hz) and, for Lobulus simplex, also in the gamma oscillation range (25–100 Hz) (McAfee et al., 2019). Furthermore, our study design lacked a direct assessment of ctACS effects on intrinsic cerebellar oscillations through EEG measurements. For future research, exploring frequencies within and beyond the theta range and incorporating EEG to measure the modulatory effect of ctACS on cerebellar oscillations will enhance the investigation of its potential in fear learning.

Even though our results show significant group differences in post-extinction US expectancy ratings and these findings might be linked to a potential loss of discrimination or fear generalization after brain stimulation, also reported in previous studies (Abend et al., 2016; Dittert et al., 2018), it has to be noted that the verum group had higher US expectancy ratings already before acquisition training. Additionally, the verum group reported significantly elevated scalp irritation throughout extinction training, possibly resulting from compromised blinding. These unpleasant skin sensations could have increased the salience of conditioned stimuli, affecting post-extinction questionnaire responses. Therefore, we approach this finding with caution to prevent potential type I errors. However, considering these issues, using topically applied local anesthetics in future experiments may still be beneficial in reducing discomfort for participants and obtaining more reliable results (McFadden et al., 2011).

## Conclusion

The present findings revealed no statistically significant differences between the verum and sham groups regarding the impact of 6 Hz ctACS on fear extinction and recall. This does not exclude cerebellar tACS effects on extinction of learned fear. Considering the involvement of cerebellar theta activity in extinction of conditioned eyeblink responses based on animal studies (Wang et al., 2014, 2019), future research could still benefit from exploring simpler fear conditioning paradigms with non-invasive brain stimulation in the theta range. Furthermore, future studies could benefit from using personalized protocols known to have enhanced precision and effectiveness in targeting specific brain oscillation patterns, compared to fixed stimulation frequencies (Del Felice et al., 2019; Baltus et al., 2020; Ayanampudi et al., 2022). Such investigations may provide clearer insights into the potential modulatory effects of non-invasive brain stimulation on fear learning and deepen our understanding of fear regulation mechanisms, potentially contributing to future therapeutic applications.

## Data availability statement

The raw data supporting the conclusions of this article will be made available by the authors, without undue reservation.

## Ethics statement

The studies involving humans were approved by the Ethics Committee of the University Hospital Essen. The studies were conducted in accordance with the local legislation and institutional requirements. The participants provided their written informed consent to participate in this study.

## Author contributions

SS: Formal Analysis, Investigation, Writing−review and editing. PZ: Investigation, Writing−review and editing. TE: Data curation, Methodology, Resources, Software, Validation, Writing−review and editing. AT: Resources, Writing−review and editing. SN: Resources, Writing−review and editing. CM: Conceptualization, Methodology, Writing−review and editing. MN: Conceptualization, Methodology, Writing−review and editing. FY: Conceptualization, Methodology, Writing−review and editing. DT: Conceptualization, Funding acquisition, Methodology, Project administration, Supervision, Writing−original draft, Writing−review and editing. GB: Conceptualization, Formal Analysis, Funding acquisition, Methodology, Project administration, Software, Supervision, Visualization, Writing−original draft, Writing−review and editing.

## Footnotes

1
http://www.R-project.org/
