# Supplementary material for: Absence of modulatory effects of 6Hz cerebellar transcranial alternating current stimulation on fear learning in men
Source: Front Hum Neurosci. 2024 Jan 9;17:1328283. doi: 10.3389/fnhum.2023.1328283 (PMC10803490; doi:10.3389/fnhum.2023.1328283)
Supplement: Supplementary file 1 [file Data_Sheet_1.PDF]

Absence of modulatory effects of 6Hz cerebellar transcranial alternating current stimulation on fear learning in men

Supplementary material

Table of contents

Supplementary data 2

Differential skin conductance responses (SCR_{diff}) 2

Initial trial analysis 2

Questionnaires 3

Supplementary table 1. Results of the non-parametric ANOVA type-statistic (ATS) for repeated measures 5

Supplementary table 2. Results of side-effects questionnaires 8

Supplementary figure 1. 9

Supplementary figure 2 10

Supplementary data

Differential skin conductance responses (SCR_{diff})

Habituation phase (day 1): The ATS revealed no significant main effects of Group (p = 0.227) or Trial (p = 0.121) or Group × Trial interaction (p = 0.325). Thus, there were no significant differences in SCR_{diff} values between the groups or across the trials.

Acquisition training (day 1): The ATS revealed a significant main effect of Trial ($F_{2.86}$ = 9.50, p = 0.002), indicating significant differences in the SCR_{diff} across the various trials. The main effect of Group (p = 0.883), and the Group × Trial interaction were not significant (p = 0.829; **Supplementary figure 1A**, **Supplementary table 1**).

Post hoc analysis of the Trial effect revealed an overall tendency for significantly higher SCR_{diff} values in the early compared to the late trials, with an overall declining trend. Specifically, SCR_{diff} values in trials 1, 5, 6, 10 and 12 were significantly higher than in trials 14 and 16 (all p values ≤ 0.037, least squares means test), trial 2 *vs.* trials 6-9 and 11-16 (all p values ≤ 0.05, least squares means test), trial 3 *vs.* trials 7-8, 11, 13-16 (all p values ≤ 0.047, least squares means test), trials 4, 7, 9, 11 and 15 *vs.* trial 16 (all p values ≤ 0.048, least squares means test).

Extinction training (day 1): The ATS revealed no significant main effects or interaction (all p values ≥ 0.418). The results indicate that there were no significant differences in the differential SCR across trials and groups during extinction training (**Supplementary figure 1A**, **Supplementary table 1**).

Recall phase (day 2): The ATS did not reveal any significant factors of Group, Trial, Context, and their interactions (all p values ≥ 0.110). These findings suggest that there were no notable variations in the differential SCR across different blocks, groups, and contexts during the recall phase of the experiment (**Supplementary figure 1A**, **Supplementary table 1**).

Initial trial analysis

Extinction training (day 1). The ANOVA-type statistics (ATS) of the SCR_{diff} showed no significant differences between the groups (p = 0.786; **Supplementary figure 1B**).

Recall (day 2). The ATS revealed a close-to-significant effect of the Context (p = 0.064), indicating marginal differences in the SCR_{diff} based on different contexts. However, there were no significant effects of Group (p = 0.899) or the interaction between Group and Context (p = 0.569) (**Supplementary figure 1B**).

Questionnaires

Valence, arousal and fear ratings. Prior to fear acquisition training, valence, arousal, and fear ratings of the $CS+_{high}$ and $CS+_{low}$ were not significantly different (all *p* values ≥ 0.462, least squares means test). After acquisition training, the valence of the $CS+_{high}$ was rated as less pleasant compared to the $CS+_{low}$. Arousal and fear towards the $CS+_{high}$ were rated higher compared to the $CS+_{low}$ (all *p* values ≥ 0.001, least squares means test). These differences persisted until the end of the experiment (all *p* values ≤ 0.041, least squares means test), with the exception of post-extinction valence ($p = 0.250$) and fear ($p = 0.073$). A non-parametric ANOVA-type statistic revealed a significant main effect of Time (all *p* values ≤ 0.001), Stimulus (all *p* values ≤ 0.001), and a Stimulus × Time interaction (all *p* values ≤ 0.001; **Supplementary figure 2A-C, Supplementary table 1).**

US unpleasantness and CS-US contingency, US Expectancy. Median unpleasantness ratings for the US were 7 (interquartile range, IQR 6-8) on a Likert scale ranging from 1 ("not unpleasant") to 9 ("very unpleasant"), with no significant differences between the groups (Mann-Whitney U test, both *p* values ≥ 0.401).

The mean reported probability of an US occurring after the presentation of the $CS+_{high}$ was estimated to be 53.24% ± 24.11%, while the mean reported probability of an US occurring after the presentation of the $CS+_{low}$ was 23.84% ± 10.78%. There were no significant differences between the reported mean probabilities in the two groups (Mann-Whitney U tests, both *p* values ≥ 0.150).

Before fear acquisition training, reported US expectancy after $CS+_{high}$ and $CS+_{low}$ did not show a significant difference ($p = 0.092$). After fear acquisition training, participants reported higher US expectation after the $CS+_{high}$ compared to the $CS+_{low}$ ($p \leq 0.001$), but not after extinction training ($p = 0.138$), however, after extinction training the verum group reported overall higher US expectancy values after both CSs compared to the sham group ($p = 0.004$). The difference between $CS+_{high}$ and $CS+_{low}$ reappeared on day 2 post-recall ($p \leq 0.001$). A non-parametric ANOVA-type statistic revealed significant main effects of Stimulus ($F_1 = 32.51$, $p \leq 0.001$), Time ($F_{2.81} = 44.25$, $p \leq 0.001$), Stimulus × Time ($F_{2.33} = 20.77$, $p \leq 0.001$), and Time × Group ($F_{2.81} = 3.55$, $p = 0.016$) interactions (**Supplementary figure 2D**, **Supplementary table 1**). *Post hoc* analysis of the Time × Group interaction revealed significant differences between pre-acquisition and post-recall US expectancy ratings the in the sham group ($p \leq 0.001$, least squares means test), but not in the verum group ($p = 0.260$, least squares means test). Moreover, there were significant post-acquisition and post-extinction differences in the sham ($p \leq 0.001$) and close to-significant

differences in the verum group (p = 0.06), primarily attributed to higher post-extinction US expectancy values in the verum group (see above). No significant differences were revealed between other time points and among groups (pre-acquisition *vs.* post-acquisition: both p values ≤ 0.001; pre-acquisition *vs.* post-extinction: both p values ≤ 0.032; post-acquisition *vs.* post-recall: both p values ≤ 0.003; post-extinction *vs.* post-recall: both p values ≥ 0.502).

Upon closer inspection of **Supplementary figure 2D**, it was apparent that pre-acquisition US expectancy values were higher in the verum group, although significant pre-acquisition group differences were not reached (p = 0.073). Therefore, we conducted an additional *post hoc* analysis with a non-parametric two-way ANOVA-type statistic to compare pre-acquisition and post-recall US expectancy values. This analysis revealed significant effects of CS and Time, as well as their interaction (all p values ≤ 0.001). However, no significant effects were found for the factor Group or other interactions (all p values ≥ 0.191). Therefore, the significant Group × Time interaction in the initial analysis was likely influenced by the overall higher pre-acquisition US expectancy values reported by the verum group.

Possible stimulation side effects. A non-parametric ANOVA-type statistic revealed a significant Group x Time interaction (F_1 = 7.83, p = 0.005), indicating that the effect varied depending on both group and time. Additionally, there was a close-to-significant effect of Time (p = 0.051), but no significant main effect of Group (p = 0.577). *Post hoc* analysis of the Group x time interaction revealed that the verum group reported significantly higher post-stimulation scalp irritation (p = 0.009, least squares means test), while the sham group did not (p = 0.418, least squares means test).

Both groups reported significantly higher post-stimulation scalp tingling, scalp itching, and fatigue compared to their respective pre-stimulation values (all p values ≤ 0.024, least squares means test). The responses to the item "increased heartrate" was significantly higher post-stimulation compared to the pre-stimulation values (p ≤ 0.001, least squares means test). Ratings of other possible stimulation side effects did not show significant differences between the groups (all p values ≥ 0.096; **Supplementary tables 1** and **2**).

Supplementary table 1. Results of the non-parametric ANOVA type-statistic (ATS) for repeated measures for skin conductance response (SCR) amplitudes, valence, arousal, fear, US expectancy and stimulation side-effect ratings comparing cerebellar verum and sham groups.

Factor	Df	*F*	*p*
**Skin conductance responses (SCRs)**			
Habituation			
Stimulus	1	21.82	**<.001***
Trial	2	10.00	**<.001***
Group	1	0.03	0.864
Stimulus × Group	1	1.05	0.305
Stimulus × Trial	1.9	2.79	0.065
Trial × Group	2	1.32	0.268
Stimulus × Trial × Group	1.9	0.58	0.552
Fear acquisition training			
Stimulus	1	37.86	**<.001***
Trial	9.46	9.46	**<.001***
Group	1	2.19	0.139
Stimulus × Group	1	0.23	0.628
Stimulus × Trial	9.37	2.35	**0.011***
Trial × Group	9.46	2.16	**0.020***
Stimulus × Trial × Group	9.37	0.84	0.584
Extinction training			
Stimulus	1	12.35	0.577
Trial	8.28	29.79	0.056
Group	1	0.43	0.670
Stimulus × Group	9.53	7.59	0.318
Stimulus × Trial	8.28	1.61	0.539
Trial × Group	1	0.12	0.678
Stimulus × Trial × Group	9.53	1.44	0.445
Recall			
Stimulus	1	0.64	0.425
Trial	2.9	13.25	**<.001***
Group	1	0.21	0.647
Context	1	1.17	0.279
Stimulus × Group	1	1.98	0.160
Stimulus × Context	1	0.30	0.586
Group × Context	1	0.13	0.720
Stimulus × Trial	3.98	1.38	0.239
Trial × Group	3.04	0.95	0.414
Trial × Context	3.95	0.20	0.937
Stimulus × Trial × Group	3.95	0.94	0.437
Stimulus × Trial × Context	4.13	1.41	0.227
Trial × Group × Context	3.98	0.74	0.563
Stimulus × Group × Context	1	0.88	0.347
Stimulus × Trial × Group × Context	3.35	0.40	0.777
**Initial trials (SCRs)**			
Extinction training			
Stimulus	1	0.94	0.332
Group	1	0.10	0.748
Stimulus × Group	1	1.13	0.288
Recall			
Stimulus	1	3.48	0.062
Group	1	0.01	0.917

Context	1	1.12	0.290
Stimulus × Group	1	<0.01	0.995
Stimulus × Context	1	6.05	**0.014***
Group × Context	1	<0.01	0.950
Stimulus × Group × Context	1	<0.01	0.988
**Differential skin conductance responses (SCR_{diff})**			
Habituation			
Trial	1.98	2.12	0.121
Group	1	1.46	0.227
Trial × Group	1.98	1.12	0.325
Fear acquisition training			
Trial	9.50	2.86	**0.002***
Group	1	0.02	0.883
Trial × Group	9.50	0.57	0.829
Extinction training			
Trial	9.58	1.02	0.418
Group	1	0.12	0.729
Trial × Group	9.58	0.57	0.833
Recall			
Trial	4.23	1.66	0.154
Group	1	2.55	0.110
Context	1	1.34	0.246
Context × Trial	4.3	1.83	0.115
Context × Group	1	1.23	0.267
Trial × Group	4.23	0.82	0.521
Context × Trial × Group	4.3	1.19	0.311
**Initial trials (SCR_{diff})**			
Extinction training			
Group	1	0.07	0.786
Recall			
Group	1	0.02	0.899
Context	1	3.44	0.064
Group × Context	1	0.32	0.569
**Valence**			
Stimulus	1	13.81	**<.001***
Time	2.77	7.46	**<.001***
Group	1	0.21	0.650
Stimulus × Time	2.15	14.52	**<.001***
Time × Group	2.77	1.11	0.343
Stimulus × Group	1	0.07	0.796
Stimulus × Time × Group	2.15	1.03	0.361
**Arousal**			
Stimulus	1	18.15	**<.001***
Time	2.44	14.92	**<.001***
Group	1	0.23	0.630
Stimulus × Time	2.73	11.78	**<.001***
Time × Group	2.44	0.99	0.385
Stimulus × Group	1	0.56	0.454
Stimulus × Time × Group	2.73	0.22	0.864
**Fear**			
Stimulus	1	10.50	**0.001***
Time	2.63	14.72	**<.001***
Group	1	0.66	0.415
Stimulus × Time	2.61	5.68	**0.001***

Time × Group	2.63	1.26	0.285
Stimulus × Group	1	0.25	0.617
Stimulus × Time × Group	2.61	1.35	0.258
	**US expectancy**		
Stimulus	1	32.51	**<.001***
Time	2.81	44.25	**<.001***
Group	1	2.86	0.091
Stimulus × Time	2.33	22.77	**<.001***
Time × Group	2.81	3.55	**0.016***
Stimulus × Group	1	0.07	0.796
Stimulus × Time × Group	2.33	0.06	0.959
	**Stimulation side-effects**		
	Headache		
Group	1	<0.01	0.953
Time	1	1.00	0.318
Group × Time	1	0.01	0.940
	Neck pain		
Group	1	0.22	0.641
Time	1	1.68	0.195
Group × Time	1	1.65	0.199
	Back pain		
Group	1	<0.01	0.955
Time	1	0.58	0.445
Group × Time	1	0.02	0.882
	Blurred vision		
Group	1	0.45	0.500
Time	1	0.48	0.491
Group × Time	1	0.11	0.736
	Scalp irritation		
Group	1	0.32	0.573
Time	1	3.80	0.051
Group × Time	1	7.83	**0.005***
	Scalp tingling		
Group	1	0.01	0.909
Time	1	5.83	**0.016***
Group × Time	1	1.98	0.160
	Scalp itching		
Group	1	0.08	0.771
Time	1	8.27	**0.004***
Group × Time	1	3.18	0.075
	Increased heartbeat		
Group	1	0.15	0.700
Time	1	13.68	**<0.001***
Group × Time	1	<0.01	0.990
	Burning sensation		
Group	1	0.68	0.411
Time	1	0.54	0.461
Group × Time	1	2.77	0.096
	Hot flashes		
Group	1	0.01	0.939
Time	1	0.30	0.582
Group × Time	1	0.23	0.635

		Vertigo	
Group	1	0.48	0.487
Time	1	0.01	0.916
Group × Time	1	0.03	0.856
		Sudden mood change	
Group	1	<0.01	0.946
Time	1	1.64	0.201
Group × Time	1	0.03	0.856
		Fatigue	
Group	1	0.36	0.549
Time	1	5.09	**0.024***
Group × Time	1	0.10	0.755
		Phosphenes	
Group	1	0.04	0.840
Time	1	0.68	0.408
Group × Time	1	0.42	0.517

* Significant results at $p < 0.05$.

Supplementary table 2. Results of side-effects questionnaires.

Self-reported median ratings and interquartile range (in brackets) on a 9-point Likert scale ranging from 1 (“*absent*”) to 9 (*“strong”*) prior and post ctACS administration.

Questionnaire item	***Prior stimulation***		***Post stimulation***	
	Sham group	***Verum group***	***Sham group***	***Verum group***
Headache	1 (1 - 1.5)	1 (1 - 1)	1 (1 - 2)	1 (1 - 1.75)
Neck pain	1 (1 - 2)	1 (1 - 3)	2 (1 - 4.5)	1 (1 - 2.75)
Back pain	1 (1 - 3)	1.5 (1 - 2)	2 (1 - 2.5)	1.5 (1 - 2.75)
Blurred vision	2 (1 - 5.5)	1 (1 - 4.75)	3 (1 - 5.5)	1.5 (1 - 3.75)
Scalp irritation	1 (1 - 1)	1 (1 - 1)	**1 (1 - 1)**†	**1 (1 - 2.75)**†
Scalp tingling	**1 (1 - 1.5)***	**1 (1 - 1)***	**1 (1 - 2.5)***	**1 (1 - 3.5)***
Scalp itching	**1 (1 - 1)***	**1 (1 - 1)***	**1 (1 - 1.5)***	**1 (1 - 2)***
Increased heartbeat	**3 (1 - 6)***	**2 (2 - 3)***	**1 (1 - 2.5)***	**1 (1 - 2)***
Burning sensation	1 (1 - 2)	1 (1 - 1)	1 (1 - 1)	1 (1 - 1)
Hot flashes	1 (1 - 1)	1 (1 - 1)	1 (1 - 1)	1 (1 - 1)
Vertigo	1 (1 - 2)	1 (1 - 1)	1 (1 - 2)	1 (1 - 1)
Sudden mood change	1 (1 - 2.5)	1.5 (1 - 2)	1 (1 - 2)	1 (1 - 2)
Fatigue	**3 (1 - 5)***	**3 (1 - 4.5)***	**3 (2.5 - 6)***	**3.5 (2 - 4.75)***
Phosphenes	1 (1 - 1)	1 (1 - 1)	1 (1 - 1)	1 (1 - 1.75)

Statistical significances (least squares means tests, $p < 0.05$) are indicated in bold.

* Significant differences between prior- and post-ctACS administration.

† Significant differences between sham and verum group.

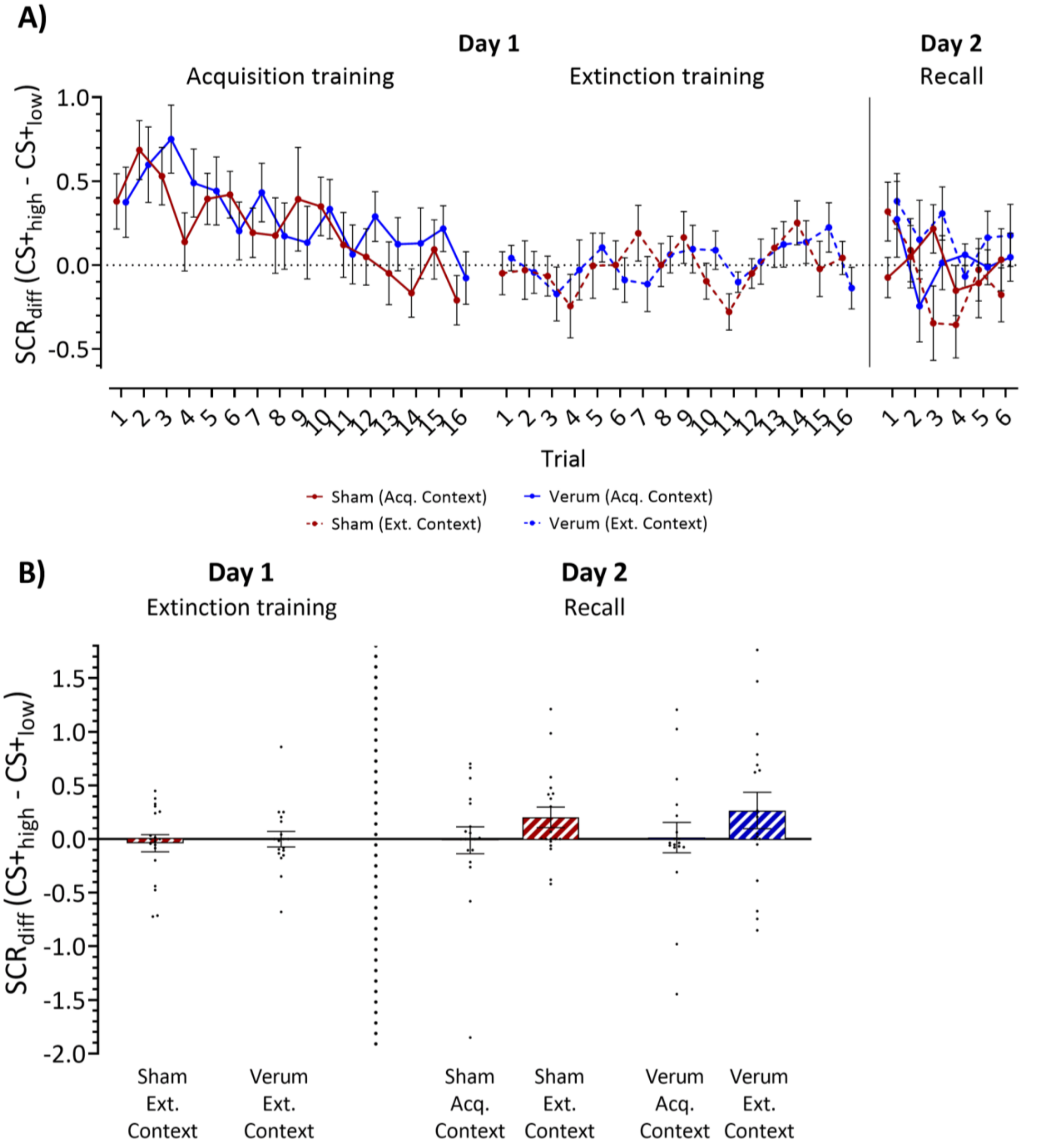

Supplementary figure 1. Log-transformed differential skin conductance response amplitudes ($CS+_{high}$ – $CS+_{low}$; SCR_{diff}). **A)** Mean SCR_{diff} values for individual trials for acquisition training, extinction training and recall phases and **B)** Recall of learned fear responses at the beginning of extinction training and recall.

A) Filled dots represent the mean values for individual trials for acquisition training, extinction training and recall phases. Solid lines connect mean values of trials presented in the acquisition context, while dotted lines connect mean values of trials presented in the extinction context. **B)** The figure shows mean SCR_{diff} averaged from the initial two trials of each phase presented in the same context. Individual responses are indicated by dots .

Error bars indicate S.E.M. Blue colors = verum, red colors = sham. CS = conditioning stimulus. Acq. Context = context presented during acquisition training, Ext. Context = context presented during extinction training.

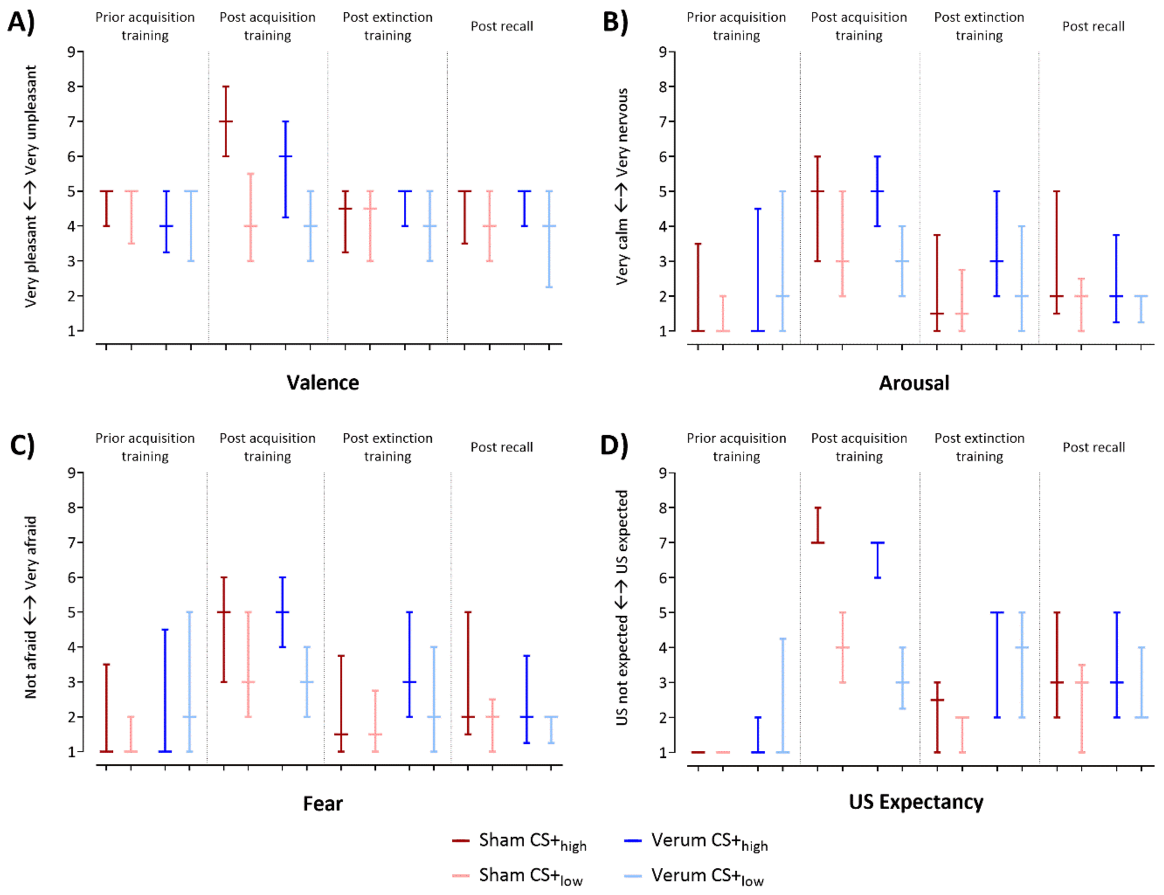

Supplementary figure 2. Median ratings for **A)** valence, **B)** arousal, **C)** fear and **D)** US expectancy obtained using a Likert-scale ranging from 1 (*"very pleasant" / "very calm" / "not afraid", "US not expected"*, respectively) to 9 (*"very unpleasant" / "very nervous" / "very afraid", "US expected"*, respectively). Median values are depicted as horizontal lines, and the whiskers represent the first to third quartile. The sham group is shown in red, the verum group in blue. CS = conditioning stimulus. Dark colors indicate $CS+_{high}$, light colors indicate $CS+_{low}$.
